# Pharmacogenetics of Antipsychotic-Induced Movement Disorders as a Resource for Better Understanding Parkinson’s Disease Modifier Genes

**DOI:** 10.3389/fneur.2015.00027

**Published:** 2015-02-20

**Authors:** Lior Greenbaum, Bernard Lerer

**Affiliations:** ^1^Department of Neurology, Sheba Medical Center at Tel Hashomer, Ramat Gan, Israel; ^2^The Joseph Sagol Neuroscience Center, Sheba Medical Center at Tel Hashomer, Ramat Gan, Israel; ^3^Biological Psychiatry Laboratory, Department of Psychiatry, Hadassah – Hebrew University Medical Center, Jerusalem, Israel

**Keywords:** Parkinson’s disease, schizophrenia, antipsychotic-induced parkinsonism, tardive dyskinesia, l-DOPA induced dyskinesia

## Abstract

Antipsychotic-induced movement disorders are major side effects of antipsychotic drugs among schizophrenia patients, and include antipsychotic-induced parkinsonism (AIP) and tardive dyskinesia (TD). Substantial pharmacogenetic work has been done in this field, and several susceptibility variants have been suggested. In this paper, the genetics of antipsychotic-induced movement disorders is considered in a broader context. We hypothesize that genetic variants that are risk factors for AIP and TD may provide insights into the pathophysiology of motor symptoms in Parkinson’s disease (PD). Since loss of dopaminergic stimulation (albeit pharmacological in AIP and degenerative in PD) is shared by the two clinical entities, genes associated with susceptibility to AIP may be modifier genes that influence clinical expression of PD motor sub-phenotypes, such as age at onset, disease severity, or rate of progression. This is due to their possible functional influence on compensatory mechanisms for striatal dopamine loss. Better compensatory potential might be beneficial at the early and later stages of the PD course. AIP vulnerability variants could also be related to latent impairment in the nigrostriatal pathway, affecting its functionality, and leading to subclinical dopaminergic deficits in the striatum. Susceptibility of PD patients to early development of l-DOPA induced dyskinesia (LID) is an additional relevant sub-phenotype. LID might share a common genetic background with TD, with which it shares clinical features. Genetic risk variants may predispose to both phenotypes, exerting a pleiotropic effect. According to this hypothesis, elucidating the genetics of antipsychotic-induced movement disorders may advance our understanding of multiple aspects of PD and it clinical course, rendering this a potentially rewarding field of study.

## Introduction

Since their introduction during the 1950s, antipsychotic drugs have been the cornerstone of pharmacological treatment for schizophrenia and other psychotic disorders (1, 2). Although all effective antipsychotics are dopamine D2 receptor antagonists (3), they are divided into two large categories, with different receptor binding and side-effect profiles: first and second generation antipsychotics (FGA, SGA) (4, 5).

Antipsychotic drugs are linked to a wide spectrum of adverse effects. FGAs cause antipsychotic-induced movement disorders, which are the focus of this paper. Antipsychotic-induced movement disorders include acute manifestations (dystonia, parkinsonism, and akathisia) as well as long-lasting effects [tardive dyskinesia (TD) and tardive dystonia] (2, 4). SGAs are linked to cardiometabolic effects (6), although movement disorder rates among SGA treated patients are not negligible and represent a prevalent clinical challenge. In the vast psychiatric literature, the term “antipsychotic-induced extrapyramidal symptoms (EPS)” is used when referring to antipsychotic-induced movement disorders. Although the meaning is the same, in this paper, we will refer to “movement disorders” instead of EPS (which is considered obsolete).

Extensive pharmacogenetic research has been performed in the field of antipsychotics (7), implementing a candidate gene approach and genome-wide association studies (GWASs) (8, 9). Regarding antipsychotic-induced movement disorders, most pharmacogenetic work has focused on TD, with modest success. Reviewing the literature of TD genetics is beyond the scope of this paper; the reader is referred to a comprehensive review by Lee and Kang (10). Briefly, candidate gene studies pointed to potential involvement of genetic variants related to dopaminergic or serotonergic transmission, drug metabolism (CYP enzyme family), and oxidative stress mechanisms (10). Several TD–GWASs have been performed, reporting some promising signals (11–14). Of particular interest is the association of the *HSPG2* SNP rs2445142 with TD, in Japanese and Caucasian populations (12, 15).

Genetic research on other types of antipsychotic-induced movement disorders is limited. Moreover, many studies regard different forms of acute drug-induced movement disorders as a single clinical entity (16). Studies that focus specifically on pharmacogenetics of antipsychotic-induced parkinsonism (AIP) are few, and have mostly concentrated on functional variants within selected candidate genes such as the dopamine receptors *DRD2* and *DRD3* and serotonin receptors *HTR2A* and *HTR2C* (16, 17). The Regulator of G protein signaling 2 (*RGS2*) gene was associated with AIP (18, 19) and supported functionally by behavioral studies of AIP like features in mice carrying a mutation leading to reduction of gene expression (20). However, other groups have failed to validate this association. (21–23). An AIP-GWAS (24) revealed association of the rs12678719 SNP in the *ZFPM2* gene with this phenotype, that was validated in an independent sample (25).

While pharmacogenetics may have major clinical value to personalized medicine in psychiatry (26, 27), the aim of this hypothesis article is to put the genetics of antipsychotic-induced movement disorders in a broader context. We suggest that genetic variants, which are risk factors for AIP and TD, may provide insights into a different disease related to the dopaminergic system, Parkinson’s disease (PD). This original hypothesis suggests that the same genes, which confer susceptibility to or protection against AIP and TD (among schizophrenia patients treated with antipsychotics), may be modifier genes that influence the clinical expression of PD in those affected by this disease. AIP variants might be related to compensatory mechanisms for dopamine loss in the striatum, or influence nigrostriatal (NS) pathway integrity and functionality and thereby possibly modulate PD motor sub-phenotypes such as age at disease onset and rate of disease progression. Another sub-phenotype of importance is susceptibility of PD patients to early development of a serious side effect of dopaminergic treatment, l-DOPA induced dyskinesia (LID). LID may share a common genetic background with TD, with which it shares clinical features.

In the following sections, we present epidemiological, imaging, and molecular data that support this line of thought. Naturally, we do not claim that AIP and motor symptoms of PD or TD and LID are clinically identical entities. On the other hand, there is substantial and intriguing similarity between two phenotype pairs, which is the background to our hypothesis. Although the empirical supporting evidence for our assumptions is limited at the current stage of the field (as presented below) and some of the underlying assumptions might be eventually wrong, the aim of this paper is to introduce a novel hypothesis and perspective, challenging future study in this direction (employing more advanced genetic, genomic, and epigenetic tools), rather than to reach definitive conclusions.

## AIP in Relation to PD Genetics

### Antipsychotic-induced parkinsonism

Antipsychotic-induced parkinsonism, the most common manifestation of antipsychotic-induced movement disorders, is an acute, reversible side effect (28, 29). AIP prevalence varies widely from 15% to more than 40% of patients treated with antipsychotics (30, 31). This substantial heterogeneity may stem from inter-study differences in medication regimens and patient demographic background. Well documented risk factors for AIP are antipsychotic type (FGA versus SGA), high doses of antipsychotics, old age, and female gender (32, 33). According to data from the 1960s, most affected patients develop AIP within the first 2 months of antipsychotic exposure, or even earlier (34, 35). Improvement occurs within a few months.

The pathophysiology of AIP is not entirely clear. As a dose dependent phenomenon, it involves occupancy of the NS pathway dopamine D2 receptor (DRD2s) (36). All known, antipsychotics are DRD2s blockers and are thought to reduce psychotic symptoms by blocking these receptors in the mesolimbic dopamine pathway (4). An undesired effect is blockade of DRD2s in the NS pathway. Occupancy of more than 80% of DRD2s, as produced by typical antipsychotics, significantly increases AIP risk, while occupancy <70% induces no AIP (31). The reduced tendency of SGAs to cause AIP may be related to their faster dissociation from DRD2s, compared to FGAs (36).

Parkinson’s disease is a common neurodegenerative disorder, characterized pathologically by selective loss of dopaminergic neurons projecting from the substantia nigra to the striatum with concomitant reduction in the striatal concentration of dopamine and accumulation of Lewy bodies (37). Disease manifestation includes motor and non-motor symptoms, variable clinical expression, and a slowly deteriorating course (38, 39). The central motor features of PD are tremor at rest, rigidity, bradykinesia, postural instability, flexed posture, and freezing of gait (40). Genetic variants considered risk factors for development of PD include common SNPs [e.g., within *MAPT* and *SNCA* (41)] and rare variants (e.g., within *LRRK2* and *GBA* genes) (42, 43), but multiple environmental risk factors were also reported (44).

The motor symptoms of AIP are similar to those of PD (45, 46). Indeed, there are clinical settings in which it is difficult to distinguish between the two (45, 47). Although AIP is considered to be more symmetric and less characterized by tremor compared than PD (48, 49), asymmetry, and tremor do occur frequently AIP patients (45, 50, 51). Other study suggested that AIP affects the upper limbs more than the lower ones (50). Naturally, reversibility of signs following antipsychotics withdrawal is characteristic of AIP and not PD. When reversibility does not happen, one should suspect underlying PD and consider DAT-SPECT examination (see below) (47). Also, while AIP is essentially a motor side effects among schizophrenia or other psychotic patients, the clinical picture of PD is much broader, and includes multiple non-motor symptoms such as neuropsychiatric disturbances (for example depression, anxiety, and hallucinations), cognitive decline, and autonomic dysfunction (52).

We suggest that genes associated with susceptibility to AIP may be modifier genes that influence the clinical expression of PD motor symptoms. The hypothesis is tethered in the fact that loss of dopaminergic stimulation (albeit reversible in AIP and irreversible in PD) is a core feature of both clinical entities. Thus, genes that affect occurrence and severity of AIP in schizophrenia patients exposed to antipsychotic-induced loss of dopaminergic stimulation, could have a similar role in modulating severity of several clinical aspects among PD patients (with neurodegenerative loss of dopaminergic NS input). Identification of gene variants that are protective against AIP (caused by administration of dopamine antagonist) could advance our understanding of the intrinsic ability of an individual to compensate for dopamine loss (best manifested in the context of PD). In addition, AIP vulnerability variants may be associated with subclinical dopaminergic deficits in the striatum that are aggravated by exposure to antipsychotics. Therefore, these variants could provide clues to the genetic contribution to NS pathway integrity in schizophrenia and PD.

### AIP genetic variants and compensatory mechanisms in PD

Parkinsonian motor signs manifest only following degeneration of 70–80% of the NS dopaminergic neuron population (53, 54). Post-mortem analysis of Parkinsonian brains indicates that extensive loss of dopamine in the putamen and caudate nucleus can be accompanied by minor clinical manifestations only (55, 56). Inter-individual difference in severity of motor symptoms during early and mid-duration PD may be attributed to existence or lack of compensatory mechanisms that counterbalance dopamine loss (57), and reduce severity of motor symptoms and progression rate (58). Several categories of potential compensatory mechanisms (in the striatum, basal ganglia, and cortical/cerebellar) have been recognized in PD [reviewed in Ref. (58, 59) and summarized in Table 1].

**Table 1:** **Examples for compensatory mechanisms categories in PD**.

**(A) Striatum localized mechanisms that maximize the effect of the remaining dopamine in this region, pre- and post-synaptic**
1. Upregulation of enzymes involved in dopamine metabolism [such as tyrosine hydroxylase (TH) or aromatic acid decarboxylase (AADC)] in order to increase dopamine synthesis in the residual neurons	(60–62)
2. Upregulation of DRD2 expression on striatal neurons (augmenting responsiveness to the remaining dopamine)	(63–65)
3. Downregulation of the dopamine transporter (DAT), which leads to decreased dopamine reuptake and higher concentration in the synaptic cleft	(62, 66, 67)
4. Increase in the number of intrinsic striatal tyrosine hydroxylase (TH) interneurons	(68–70)
**(B) Compensatory mechanisms located outside the striatum, and linked to modulation of basal ganglia direct and indirect pathways**
Reduction of globus pallidus externa inhibition by the indirect pathway, reducing PD symptom severity. This might be mediated by several neurotransmitter systems, which reduce activity of the indirect pathway (for example, enkephalins)	(58, 71)
**(C) Compensatory mechanisms positioned in brain regions and networks outside the basal ganglia but involved in motor system control**
Increased activity of relevant cortical (e.g., supplementary motor area) and cerebellar regions (counterbalancing impaired basal ganglia function)	(72–74)

If extrapolation is made from these putative compensatory mechanisms in PD to schizophrenia patients exposed to antipsychotics, lower severity of AIP symptoms seems related to the ability of the individual to counterbalance lack of dopamine induced by exogenous block of dopaminergic transmission. Individuals with higher compensatory potential would be less likely to develop AIP when treated with antipsychotics, due to mechanisms found at both striatal and extra-striatal levels, as mentioned above. Interpersonal differences in compensatory ability could be explained, at least partially, by genetic variants that are linked to one or more of the compensatory mechanisms, described in PD. However, PD pathophysiology involves loss of all dopaminergic transmission (both D1 and D2 receptor pathways), while AIP is primarily related to blockade of the DRD2 pathway. Therefore, the relevant compensatory mechanisms that effect AIP severity are probably more specifically related to DRD2 signaling at the striatal level, but may be more general and essentially similar to those mechanisms involved in PD at the extra-striatal level (e.g., alternation of indirect pathway activity by involvement of additional neurotransmission systems, or recruitment of brain region and networks in motor regions in the cortex or in the cerebellum that modulate parkinsonian symptoms and produce compensatory effect – as presented in Table 1).

If taken one step further, AIP genetic variants could be relevant for etiology of some sub-phenotypes of PD motor symptoms, resulting in early or delayed disease onset, or accelerated or retarded rate of progression. PD is a late-onset disease, and the mean age at onset (AAO) is approximately 60 years (75). However, AAO of the disease is variable, ranging from early onset cases to cases in the 8th–9th decades of life (76, 77), and several genes have been associated with PD AAO (75, 78, 79). Identification of AIP protective variants can advance understanding of the intrinsic ability of an individual to compensate for dopamine loss and delay appearance of clinical PD motor symptoms. More specifically, we suggest that AIP susceptibility variants may be associated with earlier AAO among PD patients (lower ability to compensate for dopamine loss) and AIP protective variants with delayed AAO of motor symptoms (better compensatory capacity).

In a small pilot study (Greenbaum et al., unpublished), we checked this hypothesis by genotyping several top AIP variants, and analyzing their association with PD AAO [SNPs were chosen from our AIP–GWAS and previous *RGS2* papers (18, 19, 24)]. We used two independent PD samples, recruited in Italy (*N* = 703, age range 34–76) and Israel (*N* = 233, age range 37–81). Two nominally significant associations in the expected direction were observed in the Italian sample, but did not withstand correction for multiple testing and were not validated in the Israeli sample. The negative findings do not necessarily mean that our basic assumption is incorrect. The fact that we were unable to support our hypothesis in this pilot study may stem from the relatively small sample sizes and limited statistical power. Alternatively, some of the AIP variants selected for this investigation may be false-positive due to spurious results in previous studies. If robust AIP risk variants are still undiscovered, lack of association with this PD sub-phenotype is understandable.

### Subclinical dopaminergic deficits in the NS and AIP risk variants

Turning back to schizophrenia patients, an alternative functional explanation for the role of AIP genetic risk variants may be their influence on NS pathway integrity (and therefore its ability to deliver sufficient amount of dopamine to the striatum). Dopaminergic deficits in the NS pathway may thus be a predisposing factor for AIP since schizophrenia patients with latent subclinical deficits in NS function would be more susceptible to external blockade of dopaminergic receptor, as happens in AIP (double hit model: underlying dopamine NS defect exaggerates DRD2 blockade). Thus, among AIP patients carrying the risk allele, administration of antipsychotic drugs that block DRD2s might aggravate preexisting, subclinical, pre-synaptic dopaminergic changes.

Integrity of NS tract can be evaluated by neuroimaging methods, including [^123^I]FP-CIT SPECT. The cocaine derivate [^123^I]-FP-CIT is a sensitive marker for degeneration of the NS pathway, due to its binding to the dopamine transporter (DAT), a protein localized to pre-synaptic dopaminergic terminals (80, 81). By implementing this neuroimaging technique to quantify dopaminergic deficits, it is possible to study the effects of AIP risk variants on NS integrity among healthy individuals and PD or schizophrenia patients and thereby link genetic variants to pre-synaptic dopaminergic changes. There is evidence that 33–44% of variability in pre-synaptic dopamine uptake in the striatum is influenced by genetic factors (82).

We previously examined the hypothesis that AIP risk alleles would be associated with lower FP-CIT uptake in the putamen and caudate. In a small sample of early stage, Israeli, PD patients (*N* = 49), we genotyped and analyzed the association of the *ZFPM2* rs12678719 AIP risk variant (discovered in our AIP–GWAS and validated in an additional population) with degree of NS pathway terminal degeneration, as evaluated by DAT-SPECT (25). We found that the AIP risk allele “G” was significantly associated with lower ligand uptake in the contralateral putamen (contralateral to the worst body side of parkinsonism), when controlling for disease duration (25). This implies that at least among PD patients, this variant may be linked to mechanisms that increase dopaminergic deficits in the NS pathway, a potential predisposing factor for AIP among schizophrenia patients. Interestingly, several neuroimaging studies in drug-induced parkinsonism patients showed that some of them manifest significantly diminished striatal binding in DAT scans (47, 83–85). For example, abnormal SPECT findings were found in 41 of 97 AIP affected schizophrenia patients (42%) in a recent study, raising the possibility of coexistence of NS defects combined with DRD2 blockade by antipsychotics (49). According to our hypothesis, this finding might be explained by genetic factors predisposing to the subclinical NS deficits.

Taken together, it is possible that AIP associated variants will provide insights into genetic factors influencing dopamine secretion capacity, related to NS integrity and function. This information may be valuable for understanding the preclinical stages of PD in which substantial NS degeneration exist, despite few clinical motor manifestations. Combination of existing subclinical deficits at baseline state, with ongoing neurodegenerative disease, could result in earlier AAO and faster disease progression rate.

## Possible Common Genetic Risk Factors for TD and LID

### TD and LID

Tardive dyskinesia is a chronic, severe, and potentially irreversible adverse effect of prolonged exposure to antipsychotics (36, 86). TD is characterized by abnormal involuntary movements of the face and extremities, and may fluctuate in severity (86, 87). In patients treated with FGAs, the incidence of TD is estimated as 5% per year (88), with a prevalence range of 20–25% among schizophrenia patients treated with antipsychotics chronically (86). SGAs are considered less likely to cause TD than FGAs, approximately 1% annually (89), but this rate is higher, according to other reports (90). Risk factors for TD include older age, female gender, and duration and intensity of antipsychotic treatment (91, 92). Genetic predisposition probably plays a role in determining individual TD susceptibility, as described in the introduction.

The etiology of TD is unknown, and several explanations have been suggested including supersensitivity of post-synaptic dopamine D2 receptors in the NS pathway following long-lasting blockade of dopamine (93, 94). Other possible mechanisms are damage to striatal GABA-containing neurons and dysregulation of this system (95), degeneration of striatal cholinergic interneurons (87), or neuronal damage (neurotoxicity) due to overproduction of free radicals (96, 97). Interestingly, there is evidence that spontaneous dyskinesia, similar to TD, may be present among schizophrenia patients who are antipsychotic naïve, therefore related to the disease itself (98, 99), and the prevalence increases with advancing age (100).If true, it could be that antipsychotics exposure exacerbates the manifestation of an intrinsic “motor component” of schizophrenia, which is not merely a side effect of drugs (101). TD susceptibility genes may therefore represent risk variants for a “motor prone subtype” of schizophrenia (and manifests only among minority of patients), which is related to schizophrenia pathophysiology itself, rather than pure results of drug exposure.

Moving again to PD, dopamine replacement therapy [in particular, levodopa (l-DOPA)], is regarded as the pharmacological therapy of choice for the motor symptoms of this disease (37, 40). The majority of PD patients respond well to l-DOPA during the early years of treatment (102), but most will ultimately experience disabling adverse motor manifestations such as motor fluctuations and LID. LID is involuntary movements, mostly choreiform or dystonic in nature, affecting discrete body parts (103). Once established, LID is irreversible and appears on every administration of the dopaminergic agent (104). Several classifications for LID have been purposed; the most common types are peak-dose and diphasic (105). Although the overwhelming majority of PD patients will eventually develop LID (approximately 90% after 15 years of drug exposure) (106–108), some individuals develop it early and others relatively late. An accepted estimate is that approximately 40% of PD patients will develop LID following 4–6 years of l-DOPA treatment (109). Inter-individual differences in LID susceptibility and onset-time may be explained by a combination of demographic, clinical, and genetic risk factors. Higher LID vulnerability is linked to younger age at PD onset, longer PD duration, longer duration of l-DOPA treatment, higher drug doses, and female gender (103, 110). Genetic factors may contribute to LID variability in combination with clinical risk factors.

The underlying etiology of LID is unclear. LID development first requires denervation of the basal ganglia (as happens in PD), which changes response to future dopaminergic medications (105). Then, chronic consumption of l-DOPA (or other dopaminergic agents) causes pulsatile and non-physiological stimulation of dopamine receptors in the striatum. The result is disturbance in anatomical pathways and functional mechanisms, which are responsible for proper motor control (104). This may be mediated by aberrant neuroplasticity and remodeling of relevant neuronal connectivity (104, 111). D1 receptors and their downstream signaling cascade are considered to contribute substantially to LID pathophysiology (112), although other receptors are probably also relevant [e.g., DRD3, GABA-A, adenosine A2A, and more (104, 112)].

l-DOPA induced dyskinesia and TD are both phenotypes of drug-induced hyperkinetic movement disorders, which share clinical similarities and may have an intersecting genetic background, as discussed below. Nevertheless, there are also some prominent clinical features, which differentiate the two types of dyskinesia. TD is more characterized by oral, buccal, and lingual movements (which are repetitive and complex), and limbs and trunk involvement is less severe (113). Compared to TD, LID is phenomenologically more heterogeneous, and includes chorea, athetosis, dystonia, and myoclonus (103, 114). Also, LID is pronounced in the extremities and neck (115, 116), and tend to start on the body side more severely affected by PD (113). Epidemiologically, two important and interrelated risk factors for LID susceptibility are younger age at PD onset and longer disease duration (117–119), while the most consistently established risk factor for TD is advancing age (120, 121). These differences should be kept in mind.

### TD and LID: Intersection of their genetic background

Based on the clinical and the neurobiological similarities of the two chronic drug-induced dyskinesia phenomena, we suggest a possible common genetic background shared by TD and LID, such that particular genetic variants are associated with susceptibility to TD as well as LID (same risk allele). Although the underlying disease context is different, there is a high level of clinical similarity between TD to LID, both being hyperkinetic phenotypes with uncontrolled movements. The two disorders are associated with an imbalance in the dopamine system, due to years of drug treatment leading to chronic, non-physiological, pulsatile stimulation (LID), or blockade (TD) of dopaminergic receptors. Analogy between two phenotypes may also be found at the pharmacological level; for example, overactivity of the glutamatergic system (104, 122). Amantadine, a glutamatergic antagonist, suppresses LID (123, 124) and also improves TD among schizophrenia patients (99, 125). Using MRI, volumetric abnormalities in the inferior frontal gyrus were demonstrated in patients with both phenotypes (126, 127).

Thus, we hypothesize that factors important in the pathophysiology of TD might be insightful for understanding that of LID (104). Actually, the potential existence of similar pathophysiological mechanisms between LID and TD is not new in the literature, and was postulated previously by several authors (104, 128, 129). The emphasis on the genetic aspect of this hypothesis is the novelty of this manuscript. Besides contributing to the molecular understanding of both TD and LID *per se*, this insight (if correct) may advance the field of PD pharmacogenetic as well as that of schizophrenia, due to reciprocal influences. Common genetic variants may predispose to both phenotypes. According to this risk allele sharing notion, risk variants for TD (among schizophrenia patients) may be relevant to early LID susceptibility in PD affected individuals, representing a pleiotropic effect. The extent of this genetic overlap is probably limited and partial, while additional genetic risk variants confer susceptibility to LID or TD, separately.

In a proof of concept study, we analyzed the association of 21 TD-associated SNPs with LID susceptibility in two independent samples of PD patients (130). SNPs were chosen based on previous TD–GWASs in several populations (12–14) and top results from the candidate gene literature. Controlling for relevant clinical variables, only a single SNP (*DRD2* rs1800497) was associated with LID in the Italian sample (187 early LID developers versus 203 non-early developers), in the expected direction of effect. This finding was not validated in a Jewish Israeli sample (128 LID positive and 75 negative, after 3 years of drug exposure), and did not withstand correction for multiple testing (130). However, the negative findings do not exclude the possible validity of our initial hypotheses of shared genetic predisposition (alternative explanations to lack of confirmation are elaborated above, in Section “AIP Genetic Variants and Compensatory Mechanisms in PD”). The goal of our preliminary data presentation was to demonstrate the applicability of this approach, and its possible implementation.

Compared to the relatively extensive volume of pharmacogenetic studies in TD, research on the genetics of LID is limited. Several candidate gene studies were performed in small samples [for example, Ref. (131–133)], but results are mostly inconsistent and lack replications. GWASs have not been published for LID thus far. Findings from the more mature field of TD genetics may provide reasonable candidate genetic variants for LID research (and if validated also molecular targets for further research and drug development). Analysis of novel and robust TD variants detected in the future for association with LID is indicated and may support our hypothesis. Moreover, when LID GWASs are hopefully performed and published in the future, it will be of interest to compare the top association signals from GWASs of both phenotypes, looking for overlapping risk variants and genes.

## Conclusion

Further application of pharmacogenetic tools to antipsychotic drug therapy is greatly needed (134, 135). Identification of genetic biomarkers that may assist in *a priori* prediction of antipsychotic-induced movement disorders manifestations such as AIP and TD among schizophrenia patients will be of great value. The aim of this article has been to broaden the context of antipsychotic-induced movement disorders genetics research. We put forward the hypothesis that extending our understanding of the genetic background of AIP and TD and applying this knowledge to PD could render pivotal insights into genetic and molecular aspects of PD, provide new targets for drug development, and assist in finding biomarkers for personalized medicine. We have suggested that:

- Genes, which confer susceptibility to or protection against antipsychotic-induced movement disorders (in particular, AIP and TD), might also be modifier genes that influence PD motor clinical features.
- AIP linked variants may shed light on PD sub-phenotypes such as AAO or rate of disease progression, due to their possible functional influence on compensatory mechanisms for dopamine loss in the striatum, and/or to latent deficits of the NS pathway.
- TD and LID may share genetic risk variants, associated with an uncontrolled hyperkinetic phenotype that appears following long-term fluctuating and non-physiological stimulation of striatal dopaminergic receptors.

As an unproven hypothesis, not well supported by empirical data, caution in evaluation is required. We render only modest empirical support for our hypothesis, based on traditional pharmacogenetics association studies (candidate genes and GWASs), in relatively small and probably underpowered samples. However, the aim of this paper is to raise new postulations, and stimulate direction for future research. Due to recent advances in last decade in genetics, genomics, and epigenetics research, we are now in a better position that may enable systematic investigation of these hypotheses in detail, beyond the limited tools used to date.

Larger sample GWASs will assist in this task, especially when combined with a pathway analysis approach. Since phenotype susceptibility depend on cumulative effect of variants within multiple genes in relatively small number of functional pathways (136, 137), this method may assist in shedding light on shared molecular pathways between phenotypes of interest, beyond the contribution of specific gene. Also, beneficial is the employment of functional genomics methods, which detect alternation in expression profile at the transcriptome and proteomic level, where large number of genes/proteins are analyzed simultaneously (135, 136, 138). Peripheral white blood cells and post-mortem brain tissue are potential tissue sources to discover genomic biomarkers correlated to AIP, TD, and LID. The same sources shall also be used in investigation of shared epigenetics mechanisms to phenotypes of interest, such as DNA methylation or micro RNA (e.g., epigenome-wide association studies) (43, 139). Last, multiple rare genetic variants with large effect size (discovery by whole exome sequencing) may also be relevant to genetics of antipsychotic-induced movement disorders, and its intersection with PD. Currently, understanding the contribution of rare variants to pharmacogenetic traits is only at the beginning. Integration of data derived from these approaches, in addition to animal models studies (available for most phenotypes discussed here), aiming to gain mechanistic insights – will assist in better understanding of the genetic architecture of the phenotypes discussed above, and the potential intersection between them.

As novel findings and mechanisms will emerge and accumulate, it will be possible to validate the hypothesis presented, or refute it. However, if these assumptions are correct, even partially, then the incentive for deciphering antipsychotic-induced movement disorders genetic is high, with broader applications for PD and schizophrenia research.

## Author Contributions

LG and BL jointly developed the concept of this article, reviewed the relevant literature, and wrote the manuscript.

## Conflict of Interest Statement

The authors declare that the research was conducted in the absence of any commercial or financial relationships that could be construed as a potential conflict of interest.

## References

[1] Lieberman JA Stroup TS McEvoy JP Swartz MS Rosenheck RA Perkins DO Effectiveness of antipsychotic drugs in patients with chronic schizophrenia. N Engl J Med (2005) 353(12):1209–23 10.1056/NEJMoa051688 16172203

[2] Stahl SM Stahl’s Essential Psychopharmacology Neuroscientific Basis and Practical Applications. 3rd ed New York: Cambridge university press (2008).

[3] Arranz MJ Rivera M Munro JC. Pharmacogenetics of response to antipsychotics in patients with schizophrenia. CNS Drugs (2011) 25(11):933–69. 10.2165/11595380-000000000-00000 22054119

[4] Miyamoto S Duncan GE Marx CE Lieberman JA. Treatments for schizophrenia: a critical review of pharmacology and mechanisms of action of antipsychotic drugs. Mol Psychiatry (2005) 10(1):79–104. 10.1038/sj.mp.4001556 15289815

[5] Ellenbroek BA. Psychopharmacological treatment of schizophrenia: what do we have, and what could we get? Neuropharmacology (2012) 62(3):1371–80. 10.1016/j.neuropharm.2011.03.013 21420988

[6] Fenton WS Chavez MR Medication-induced weight gain and dyslipidemia in patients with schizophrenia. Am J Psychiatry (2006) 163(10):1697–704 10.1176/ajp.2006.163.10.1697 17012676

[7] Cacabelos R Hashimoto R Takeda M. Pharmacogenomics of antipsychotics efficacy for schizophrenia. Psychiatry Clin Neurosci (2011) 65(1):3–19. 10.1111/j.1440-1819.2010.02168.x 21265934

[8] Chowdhury NI Remington G Kennedy JL. Genetics of antipsychotic-induced side effects and agranulocytosis. Curr Psychiatry Rep (2011) 13(2):156–65. 10.1007/s11920-011-0185-3 21336863

[9] Muller DJ Chowdhury NI Zai CC The pharmacogenetics of antipsychotic-induced adverse events. Curr Opin Psychiatry (2013) 26(2):144–50 10.1097/YCO.0b013e32835dc9da 23370274

[10] Lee HJ Kang SG. Genetics of tardive dyskinesia. Int Rev Neurobiol (2011) 98:231–64. 10.1016/B978-0-12-381328-2.00010-9 21907090

[11] Aberg K Adkins DE Bukszar J Webb BT Caroff SN Miller DD Genomewide association study of movement-related adverse antipsychotic effects. Biol Psychiatry (2010) 67(3):279–82. 10.1016/j.biopsych.2009.08.036 19875103 PMC3388725

[12] Syu A Ishiguro H Inada T Horiuchi Y Tanaka S Ishikawa M Association of the HSPG2 gene with neuroleptic-induced tardive dyskinesia. Neuropsychopharmacology (2010) 35(5):1155–64. 10.1038/npp.2009.220 20072119 PMC3055411

[13] Greenbaum L Alkelai A Rigbi A Kohn Y Lerer B. Evidence for association of the GLI2 gene with tardive dyskinesia in patients with chronic schizophrenia. Mov Disord (2010) 25(16):2809–17. 10.1002/mds.23377 20939080

[14] Tanaka S Syu A Ishiguro H Inada T Horiuchi Y Ishikawa M DPP6 as a candidate gene for neuroleptic-induced tardive dyskinesia. Pharmacogenomics J (2013) 13(1):27–34. 10.1038/tpj.2011.36 21826085

[15] Greenbaum L Alkelai A Zozulinsky P Kohn Y Lerer B. Support for association of HSPG2 with tardive dyskinesia in Caucasian populations. Pharmacogenomics J (2012) 12(6):513–20. 10.1038/tpj.2011.32 21808285

[16] Al Hadithy AF Wilffert B Stewart RE Looman NM Bruggeman R Brouwers JR Pharmacogenetics of parkinsonism, rigidity, rest tremor, and bradykinesia in African-Caribbean inpatients: differences in association with dopamine and serotonin receptors. Am J Med Genet B Neuropsychiatr Genet (2008) 147B(6):890–7. 10.1002/ajmg.b.30746 18389501

[17] Gunes A Dahl ML Spina E Scordo MG. Further evidence for the association between 5-HT2C receptor gene polymorphisms and extrapyramidal side effects in male schizophrenic patients. Eur J Clin Pharmacol (2008) 64(5):477–82. 10.1007/s00228-007-0450-x 18205001

[18] Greenbaum L Strous RD Kanyas K Merbl Y Horowitz A Karni O Association of the RGS2 gene with extrapyramidal symptoms induced by treatment with antipsychotic medication. Pharmacogenet Genomics (2007) 17(7):519–28. 10.1097/FPC.0b013e32800ffbb4 17558307

[19] Greenbaum L Smith RC Rigbi A Strous R Teltsh O Kanyas K Further evidence for association of the RGS2 gene with antipsychotic-induced parkinsonism: protective role of a functional polymorphism in the 3’-untranslated region. Pharmacogenomics J (2009) 9(2):103–10. 10.1038/tpj.2008.6 18347610

[20] Greenbaum L Lifschytz T Zozulinsky P Broner EC Slonimsky A Kohn Y Alteration in RGS2 expression level is associated with changes in haloperidol induced extrapyramidal features in a mutant mouse model. Eur Neuropsychopharmacol (2012) 22(5):379–86. 10.1016/j.euroneuro.2011.09.006 21982117

[21] Al Hadithy AF Wilffert B Bruggeman R Stewart RE Brouwers JR Matroos GE Lack of association between antipsychotic-induced parkinsonism or its subsymptoms and rs4606 SNP of RGS2 gene in African-Caribbeans and the possible role of the medication: the Curacao extrapyramidal syndromes study X. Hum Psychopharmacol (2009) 24(2):123–8. 10.1002/hup.997 19156702

[22] Higa M Ohnuma T Maeshima H Hatano T Hanzawa R Shibata N Association analysis between functional polymorphism of the rs4606 SNP in the RGS2 gene and antipsychotic-induced parkinsonism in Japanese patients with schizophrenia: results from the Juntendo University Schizophrenia Projects (JUSP). Neurosci Lett (2010) 469(1):55–9. 10.1016/j.neulet.2009.11.043 19931593

[23] Bakker PR Bakker E Amin N van Duijn CM van Os J van Harten PN. Candidate gene-based association study of antipsychotic-induced movement disorders in long-stay psychiatric patients: a prospective study. PLoS One (2012) 7(5):e36561. 10.1371/journal.pone.0036561 22615781 PMC3352907

[24] Alkelai A Greenbaum L Rigbi A Kanyas K Lerer B. Genome-wide association study of antipsychotic-induced parkinsonism severity among schizophrenia patients. Psychopharmacology (Berl) (2009) 206(3):491–9. 10.1007/s00213-009-1627-z 19680635

[25] Greenbaum L Smith RC Lorberboym M Alkelai A Zozulinsky P Lifschytz T Association of the ZFPM2 gene with antipsychotic-induced parkinsonism in schizophrenia patients. Psychopharmacology (Berl) (2012) 220(3):519–28. 10.1007/s00213-011-2499-6 21947317

[26] Lerer B Segman RH. Pharmacogenetics of antipsychotic therapy: pivotal research issues and the prospects for clinical implementation. Dialogues Clin Neurosci (2006) 8(1):85–94. 16640118 10.31887/DCNS.2006.8.1/blerer PMC3181755

[27] Zhang JP Malhotra AK. Pharmacogenetics of antipsychotics: recent progress and methodological issues. Expert Opin Drug Metab Toxicol (2013) 9(2):183–91. 10.1517/17425255.2013.736964 23199282 PMC3547146

[28] Rochon PA Stukel TA Sykora K Gill S Garfinkel S Anderson GM Atypical antipsychotics and parkinsonism. Arch Intern Med (2005) 165(16):1882–8. 10.1001/archinte.165.16.1882 16157833

[29] Tenback DE van Harten PN Slooff CJ van Os J. Evidence that early extrapyramidal symptoms predict later tardive dyskinesia: a prospective analysis of 10,000 patients in the European schizophrenia outpatient health outcomes (SOHO) study. Am J Psychiatry (2006) 163(8):1438–40. 10.1176/appi.ajp.163.8.1438 16877660

[30] Hansen TE Casey DE Hoffman WF. Neuroleptic intolerance. Schizophr Bull (1997) 23(4):567–82. 10.1093/schbul/23.4.567 9365996

[31] Hirose G Drug induced parkinsonism: a review. J Neurol (2006) 253(Suppl 3):III/22–III/4.

[32] Ebadi M Srinivasan SK Pathogenesis, prevention, and treatment of neuroleptic-induced movement disorders. Pharmacol Rev (1995) 47(4): 575–604. 8746555

[33] Caroff SN Hurford I Lybrand J Campbell EC Movement disorders induced by antipsychotic drugs: implications of the CATIE schizophrenia trial. Neurol Clin (2011) 29(1):127–48 10.1016/j.ncl.2010.10.002 21172575 PMC3018852

[34] Ayd FJ Jr A survey of drug-induced extrapyramidal reactions. JAMA (1961) 175:1054–60 10.1001/jama.1961.03040120016004 13685365

[35] Freyhan FA Therapeutic implications of differential effects of new phenothiazine compounds. Am J Psychiatry (1959) 115(7):577–85 10.1176/ajp.115.7.577 13617475

[36] Casey DE. Pathophysiology of antipsychotic drug-induced movement disorders. J Clin Psychiatry (2004) 65(Suppl 9):25–8. 15189109

[37] Lees AJ Hardy J Revesz T. Parkinson’s disease. Lancet (2009) 373(9680):2055–66. 10.1016/S0140-6736(09)60492-X 19524782

[38] Dauer W Przedborski S Parkinson’s disease: mechanisms and models. Neuron (2003) 39(6):889–909 10.1016/S0896-6273(03)00568-3 12971891

[39] Obeso JA Rodriguez-Oroz MC Goetz CG Marin C Kordower JH Rodriguez M Missing pieces in the Parkinson’s disease puzzle. Nat Med (2010) 16(6):653–61. 10.1038/nm.2165 20495568

[40] Fahn S. How do you treat motor complications in Parkinson’s disease: medicine, surgery, or both? Ann Neurol (2008) 64(Suppl 2):S56–64. 10.1002/ana.21453 19127577

[41] Klein C Westenberger A. Genetics of Parkinson’s disease. Cold Spring Harb Perspect Med (2012) 2(1):a008888. 10.1101/cshperspect.a008888 22315721 PMC3253033

[42] Shulman JM De Jager PL Feany MB Parkinson’s disease: genetics and pathogenesis. Annu Rev Pathol (2011) 6:193–222 10.1146/annurev-pathol-011110-130242 21034221

[43] Coppede F. Genetics and epigenetics of Parkinson’s disease. ScientificWorldJournal (2012) 2012:489830. 10.1100/2012/489830 22623900 PMC3353471

[44] Noyce AJ Bestwick JP Silveira-Moriyama L Hawkes CH Giovannoni G Lees AJ Meta-analysis of early nonmotor features and risk factors for Parkinson disease. Ann Neurol (2012) 72(6):893–901. 10.1002/ana.23687 23071076 PMC3556649

[45] Hardie RJ Lees AJ. Neuroleptic-induced Parkinson’s syndrome: clinical features and results of treatment with levodopa. J Neurol Neurosurg Psychiatry (1988) 51(6):850–4. 10.1136/jnnp.51.6.850 2900293 PMC1033159

[46] Susatia F Fernandez HH. Drug-induced parkinsonism. Curr Treat Options Neurol (2009) 11(3):162–9. 10.1007/s11940-009-0019-3 19364450

[47] Lorberboym M Treves TA Melamed E Lampl Y Hellmann M Djaldetti R. [123I]-FP/CIT SPECT imaging for distinguishing drug-induced parkinsonism from Parkinson’s disease. Mov Disord (2006) 21(4):510–4. 10.1002/mds.20748 16250023

[48] Hausner RS Neuroleptic-induced parkinsonism and Parkinson’s disease: differential diagnosis and treatment. J Clin Psychiatry (1983) 44(1):13–6. 6130081

[49] Tinazzi M Cipriani A Matinella A Cannas A Solla P Nicoletti A [(1)(2)(3)I]FP-CIT single photon emission computed tomography findings in drug-induced parkinsonism. Schizophr Res (2012) 139(1–3):40–5. 10.1016/j.schres.2012.06.003 22727453

[50] Hassin-Baer S Sirota P Korczyn AD Treves TA Epstein B Shabtai H Clinical characteristics of neuroleptic-induced parkinsonism. J Neural Transm (2001) 108(11):1299–308. 10.1007/s007020100006 11768628

[51] Djaldetti R Ziv I Melamed E. The mystery of motor asymmetry in Parkinson’s disease. Lancet Neurol (2006) 5(9):796–802. 10.1016/S1474-4422(06)70549-X 16914408

[52] Jankovic J Parkinson’s disease: clinical features and diagnosis. J Neurol Neurosurg Psychiatry (2008) 79(4):368–76 10.1136/jnnp.2007.131045 18344392

[53] Perez XA Parameswaran N Huang LZ O’Leary KT Quik M. Pre-synaptic dopaminergic compensation after moderate nigrostriatal damage in non-human primates. J Neurochem (2008) 105(5):1861–72. 10.1111/j.1471-4159.2008.05268.x 18248617 PMC3264543

[54] de la Fuente-Fernandez R Schulzer M Kuramoto L Cragg J Ramachandiran N Au WL Age-specific progression of nigrostriatal dysfunction in Parkinson’s disease. Ann Neurol (2011) 69(5):803–10. 10.1002/ana.22284 21246604

[55] Agid Y Parkinson’s disease: pathophysiology. Lancet (1991) 337(8753):1321–4 10.1016/0140-6736(91)92989-F 1674304

[56] Bernheimer H Birkmayer W Hornykiewicz O Jellinger K Seitelberger F Brain dopamine and the syndromes of Parkinson and Huntington. Clinical, morphological and neurochemical correlations. J Neurol Sci (1973) 20(4):415–55 10.1016/0022-510X(73)90175-5 4272516

[57] Obeso JA Rodriguez-Oroz MC Lanciego JL Rodriguez Diaz M How does Parkinson’s disease begin? The role of compensatory mechanisms. Trends Neurosci (2004) 27(3):125–7 10.1016/j.tins.2003.12.006 15036875

[58] Brotchie J Fitzer-Attas C. Mechanisms compensating for dopamine loss in early Parkinson disease. Neurology (2009) 72(7 Suppl):S32–8. 10.1212/WNL.0b013e318198e0e9 19221312

[59] Greenbaum L Lorberboym M Melamed E Rigbi A Barhum Y Kohn Y Perspective: identification of genetic variants associated with dopaminergic compensatory mechanisms in early Parkinson’s disease. Front Neurosci (2013) 7:52. 10.3389/fnins.2013.00052 23596382 PMC3625833

[60] Zigmond MJ Abercrombie ED Berger TW Grace AA Stricker EM. Compensations after lesions of central dopaminergic neurons: some clinical and basic implications. Trends Neurosci (1990) 13(7):290–6. 10.1016/0166-2236(90)90112-N 1695406

[61] Zigmond MJ Acheson AL Stachowiak MK Stricker EM. Neurochemical compensation after nigrostriatal bundle injury in an animal model of preclinical parkinsonism. Arch Neurol (1984) 41(8):856–61. 10.1001/archneur.1984.04050190062015 6147127

[62] Lee CS Samii A Sossi V Ruth TJ Schulzer M Holden JE In vivo positron emission tomographic evidence for compensatory changes in presynaptic dopaminergic nerve terminals in Parkinson’s disease. Ann Neurol (2000) 47(4):493–503. 10.1002/1531-8249(200004)47:4<493::AID-ANA13>3.3.CO;2-W 10762161

[63] Creese I Burt DR Snyder SH. Dopamine receptor binding enhancement accompanies lesion-induced behavioral supersensitivity. Science (1977) 197(4303):596–8. 10.1126/science.877576 877576

[64] Falardeau P Bedard PJ Di Paolo T. Relation between brain dopamine loss and D2 dopamine receptor density in MPTP monkeys. Neurosci Lett (1988) 86(2):225–9. 10.1016/0304-3940(88)90575-7 2966905

[65] Todd RD Carl J Harmon S O’Malley KL Perlmutter JS. Dynamic changes in striatal dopamine D2 and D3 receptor protein and mRNA in response to 1-methyl-4-phenyl-1,2,3,6-tetrahydropyridine (MPTP) denervation in baboons. J Neurosci (1996) 16(23):7776–82. 8922433 10.1523/JNEUROSCI.16-23-07776.1996 PMC6579075

[66] Uhl GR Walther D Mash D Faucheux B Javoy-Agid F. Dopamine transporter messenger RNA in Parkinson’s disease and control substantia nigra neurons. Ann Neurol (1994) 35(4):494–8. 10.1002/ana.410350421 8154880

[67] Sossi V de la Fuente-Fernandez R Schulzer M Troiano AR Ruth TJ Stoessl AJ. Dopamine transporter relation to dopamine turnover in Parkinson’s disease: a positron emission tomography study. Ann Neurol (2007) 62(5):468–74. 10.1002/ana.21204 17886297

[68] Porritt MJ Batchelor PE Hughes AJ Kalnins R Donnan GA Howells DW New dopaminergic neurons in Parkinson’s disease striatum. Lancet (2000) 356(9223):44–5 10.1016/S0140-6736(00)02437-5 10892768

[69] Huot P Levesque M Parent A. The fate of striatal dopaminergic neurons in Parkinson’s disease and Huntington’s chorea. Brain (2007) 130(Pt 1):222–32. 10.1093/brain/awl332 17142832

[70] Huot P Levesque M Morissette M Calon F Dridi M Di Paolo T L-DOPA treatment abolishes the numerical increase in striatal dopaminergic neurons in parkinsonian monkeys. J Chem Neuroanat (2008) 35(1):77–84. 10.1016/j.jchemneu.2007.06.004 17706922

[71] Black KJ Koller JM Campbell MC Gusnard DA Bandak SI. Quantification of indirect pathway inhibition by the adenosine A2a antagonist SYN115 in Parkinson disease. J Neurosci (2010) 30(48):16284–92. 10.1523/JNEUROSCI.2590-10.2010 21123574 PMC3008651

[72] Palmer SJ Ng B Abugharbieh R Eigenraam L McKeown MJ. Motor reserve and novel area recruitment: amplitude and spatial characteristics of compensation in Parkinson’s disease. Eur J Neurosci (2009) 29(11):2187–96. 10.1111/j.1460-9568.2009.06753.x 19490021

[73] Yu H Sternad D Corcos DM Vaillancourt DE. Role of hyperactive cerebellum and motor cortex in Parkinson’s disease. Neuroimage (2007) 35(1):222–33. 10.1016/j.neuroimage.2006.11.047 17223579 PMC1853309

[74] Appel-Cresswell S de la Fuente-Fernandez R Galley S McKeown MJ Imaging of compensatory mechanisms in Parkinson’s disease. Curr Opin Neurol (2010) 23(4):407–12 10.1097/WCO.0b013e32833b6019 20610991

[75] Latourelle JC Pankratz N Dumitriu A Wilk JB Goldwurm S Pezzoli G Genomewide association study for onset age in Parkinson disease. BMC Med Genet (2009) 10:98. 10.1186/1471-2350-10-98 19772629 PMC2758866

[76] Bower JH Maraganore DM McDonnell SK Rocca WA. Incidence and distribution of parkinsonism in Olmsted County, Minnesota, 1976-1990. Neurology (1999) 52(6):1214–20. 10.1212/WNL.52.6.1214 10214746

[77] Schrag A Schott JM. Epidemiological, clinical, and genetic characteristics of early-onset parkinsonism. Lancet Neurol (2006) 5(4):355–63. 10.1016/S1474-4422(06)70411-2 16545752

[78] Greenbaum L Rigbi A Lipshtat N Cilia R Tesei S Asselta R Association of nicotine dependence susceptibility gene, CHRNA5, with Parkinson’s disease age at onset: gene and smoking status interaction. Parkinsonism Relat Disord (2013) 19(1):72–6. 10.1016/j.parkreldis.2012.07.007 22884254

[79] Klebe S Golmard JL Nalls MA Saad M Singleton AB Bras JM The Val158Met COMT polymorphism is a modifier of the age at onset in Parkinson’s disease with a sexual dimorphism. J Neurol Neurosurg Psychiatry (2013) 84(6):666–73. 10.1136/jnnp-2012-304475 23408064 PMC3646288

[80] Booij J Knol RJ. SPECT imaging of the dopaminergic system in (premotor) Parkinson’s disease. Parkinsonism Relat Disord (2007) 13(Suppl 3):S425–8. 10.1016/S1353-8020(08)70042-7 18267276

[81] Varrone A Halldin C. Molecular imaging of the dopamine transporter. J Nucl Med (2010) 51(9):1331–4. 10.2967/jnumed.109.065656 20720060

[82] Stokes PR Shotbolt P Mehta MA Turkheimer E Benecke A Copeland C Nature or nurture? Determining the heritability of human striatal dopamine function: an [18F]-DOPA PET study. Neuropsychopharmacology (2013) 38(3):485–91. 10.1038/npp.2012.207 23093224 PMC3547199

[83] Burn DJ Brooks DJ. Nigral dysfunction in drug-induced parkinsonism: an 18F-dopa PET study. Neurology (1993) 43(3 Pt 1):552–6. 10.1212/WNL.43.3_Part_1.552 8451000

[84] Tinazzi M Ottaviani S Isaias IU Pasquin I Steinmayr M Vampini C [123I]FP-CIT SPET imaging in drug-induced parkinsonism. Mov Disord (2008) 23(13):1825–9. 10.1002/mds.22098 18759353

[85] Tinazzi M Antonini A Bovi T Pasquin I Steinmayr M Moretto G Clinical and [123I]FP-CIT SPET imaging follow-up in patients with drug-induced parkinsonism. J Neurol (2009) 256(6):910–5. 10.1007/s00415-009-5039-0 19252795

[86] Blanchet PJ. Antipsychotic drug-induced movement disorders. Can J Neurol Sci (2003) 30(Suppl 1):S101–7. 10.1017/S0317167100003309 12691483

[87] Margolese HC Chouinard G Kolivakis TT Beauclair L Miller R. Tardive dyskinesia in the era of typical and atypical antipsychotics. Part 1: pathophysiology and mechanisms of induction. Can J Psychiatry (2005) 50(9):541–7. 16262110 10.1177/070674370505000907

[88] Glazer WM Morgenstern H Doucette JT. Predicting the long-term risk of tardive dyskinesia in outpatients maintained on neuroleptic medications. J Clin Psychiatry (1993) 54(4):133–9. 8098030

[89] Remington G. Tardive dyskinesia: eliminated, forgotten, or overshadowed? Curr Opin Psychiatry (2007) 20(2):131–7. 10.1097/YCO.0b013e328017f6b1 17278910

[90] Correll CU Schenk EM Tardive dyskinesia and new antipsychotics. Curr Opin Psychiatry (2008) 21(2):151–6 10.1097/YCO.0b013e3282f53132 18332662

[91] Yassa R Jeste DV. Gender differences in tardive dyskinesia: a critical review of the literature. Schizophr Bull (1992) 18(4):701–15. 10.1093/schbul/18.4.701 1359633

[92] Kane JM Tardive dyskinesia: epidemiological and clinical presentation. In: Bloom FE Kupfer DJ, editors. Psychopharmacology: The 4th Generation of Progress. New York, NY: Raven Press (1995). p. 1485–96.

[93] Barnes TR McPhillips MA. Novel antipsychotics, extrapyramidal side effects and tardive dyskinesia. Int Clin Psychopharmacol (1998) 13(Suppl 3):S49–57. 10.1097/00004850-199803003-00009 9690971

[94] Egan MF Apud J Wyatt RJ Treatment of tardive dyskinesia. Schizophr Bull (1997) 23(4):583–609 10.1093/schbul/23.4.583 9365997

[95] Delfs JM Ellison GD Mercugliano M Chesselet MF. Expression of glutamic acid decarboxylase mRNA in striatum and pallidum in an animal model of tardive dyskinesia. Exp Neurol (1995) 133(2):175–88. 10.1006/exnr.1995.1020 7544289

[96] Sagara Y. Induction of reactive oxygen species in neurons by haloperidol. J Neurochem (1998) 71(3):1002–12. 10.1046/j.1471-4159.1998.71031002.x 9721725

[97] Naidu PS Singh A Kulkarni SK. Carvedilol attenuates neuroleptic-induced orofacial dyskinesia: possible antioxidant mechanisms. Br J Pharmacol (2002) 136(2):193–200. 10.1038/sj.bjp.0704717 12010767 PMC1573352

[98] Pappa S Dazzan P. Spontaneous movement disorders in antipsychotic-naive patients with first-episode psychoses: a systematic review. Psychol Med (2009) 39(7):1065–76. 10.1017/S0033291708004716 19000340

[99] Konig P Chwatal K Havelec L Riedl F Schubert H Schultes H. Amantadine versus biperiden: a double-blind study of treatment efficacy in neuroleptic extrapyramidal movement disorders. Neuropsychobiology (1996) 33(2):80–4. 10.1159/000119254 8927233

[100] Fenton WS Prevalence of spontaneous dyskinesia in schizophrenia. J Clin Psychiatry (2000) 61(Suppl 4):10–4. 10739325

[101] Whitty PF Owoeye O Waddington JL. Neurological signs and involuntary movements in schizophrenia: intrinsic to and informative on systems pathobiology. Schizophr Bull (2009) 35(2):415–24. 10.1093/schbul/sbn126 18791074 PMC2659305

[102] Mercuri NB Bernardi G. The ‘magic’ of L-DOPA: why is it the gold standard Parkinson’s disease therapy? Trends Pharmacol Sci (2005) 26(7):341–4. 10.1016/j.tips.2005.05.002 15936832

[103] Prashanth LK Fox S Meissner WG. L-DOPA-induced dyskinesia-clinical presentation, genetics, and treatment. Int Rev Neurobiol (2011) 98:31–54. 10.1016/B978-0-12-381328-2.00002-X 21907082

[104] Jenner P. Molecular mechanisms of L-DOPA-induced dyskinesia. Nat Rev Neurosci (2008) 9(9):665–77. 10.1038/nrn2471 18714325

[105] Del Sorbo F Albanese A. Levodopa-induced dyskinesias and their management. J Neurol (2008) 255(Suppl 4):32–41. 10.1007/s00415-008-4006-5 18821084

[106] Rascol O Brooks DJ Korczyn AD De Deyn PP Clarke CE Lang AE. A five-year study of the incidence of dyskinesia in patients with early Parkinson’s disease who were treated with ropinirole or levodopa. 056 Study Group. N Engl J Med (2000) 342(20):1484–91. 10.1056/NEJM200005183422004 10816186

[107] Hely MA Morris JG Reid WG Trafficante R. Sydney multicenter study of Parkinson’s disease: non-L-DOPA-responsive problems dominate at 15 years. Mov Disord (2005) 20(2):190–9. 10.1002/mds.20324 15551331

[108] Fabbrini G Brotchie JM Grandas F Nomoto M Goetz CG. Levodopa-induced dyskinesias. Mov Disord (2007) 22(10):1379–89. 10.1002/mds.21475 17427940

[109] Ahlskog JE Muenter MD. Frequency of levodopa-related dyskinesias and motor fluctuations as estimated from the cumulative literature. Mov Disord (2001) 16(3):448–58. 10.1002/mds.1090 11391738

[110] Zappia M Annesi G Nicoletti G Arabia G Annesi F Messina D Sex differences in clinical and genetic determinants of levodopa peak-dose dyskinesias in Parkinson disease: an exploratory study. Arch Neurol (2005) 62(4):601–5. 10.1001/archneur.62.4.601 15824260

[111] Olanow CW Obeso JA Stocchi F. Continuous dopamine-receptor treatment of Parkinson’s disease: scientific rationale and clinical implications. Lancet Neurol (2006) 5(8):677–87. 10.1016/S1474-4422(06)70521-X 16857573

[112] Fisone G Bezard E Molecular mechanisms of L-DOPA-induced dyskinesia. Int Rev Neurobiol (2011) 98:95–122 10.1016/B978-0-12-381328-2.00004-3 21907084

[113] Ha AD Jankovic J. An introduction to dyskinesia – the clinical spectrum. Int Rev Neurobiol (2011) 98:1–29. 10.1016/B978-0-12-381328-2.00001-8 21907081

[114] Fahn S The spectrum of levodopa-induced dyskinesias. Ann Neurol (2000) 47(4 Suppl 1):S2–9. 10762127

[115] Fabbrini G Defazio G Colosimo C Suppa A Bloise M Berardelli A. Onset and spread of dyskinesias and motor symptoms in Parkinson’s disease. Mov Disord (2009) 24(14):2091–6. 10.1002/mds.22703 19691126

[116] Jankovic J. Motor fluctuations and dyskinesias in Parkinson’s disease: clinical manifestations. Mov Disord (2005) 20(Suppl 11):S11–6. 10.1002/mds.20458 15822109

[117] Tambasco N Simoni S Marsili E Sacchini E Murasecco D Cardaioli G Clinical aspects and management of levodopa-induced dyskinesia. Parkinsons Dis (2012) 2012:745947. 10.1155/2012/745947 22701811 PMC3372050

[118] Kostic V Przedborski S Flaster E Sternic N. Early development of levodopa-induced dyskinesias and response fluctuations in young-onset Parkinson’s disease. Neurology (1991) 41(2 Pt 1):202–5. 10.1212/WNL.41.2_Part_1.202 1992362

[119] Schrag A Quinn N Dyskinesias and motor fluctuations in Parkinson’s disease. A community-based study. Brain (2000) 123(Pt 11):2297–305. 11050029 10.1093/brain/123.11.2297

[120] Woerner MG Alvir JM Saltz BL Lieberman JA Kane JM. Prospective study of tardive dyskinesia in the elderly: rates and risk factors. Am J Psychiatry (1998) 155(11):1521–8. 10.1176/ajp.155.11.1521 9812112

[121] Yassa R Nastase C Dupont D Thibeau M. Tardive dyskinesia in elderly psychiatric patients: a 5-year study. Am J Psychiatry (1992) 149(9):1206–11. 10.1176/ajp.149.9.1206 1354413

[122] Tsai G Goff DC Chang RW Flood J Baer L Coyle JT. Markers of glutamatergic neurotransmission and oxidative stress associated with tardive dyskinesia. Am J Psychiatry (1998) 155(9):1207–13. 10.1176/ajp.155.9.1207 9734544

[123] Blanchet PJ Konitsiotis S Chase TN. Amantadine reduces levodopa-induced dyskinesias in parkinsonian monkeys. Mov Disord (1998) 13(5):798–802. 10.1002/mds.870130507 9756148

[124] Del Dotto P Pavese N Gambaccini G Bernardini S Metman LV Chase TN Intravenous amantadine improves levadopa-induced dyskinesias: an acute double-blind placebo-controlled study. Mov Disord (2001) 16(3):515–20. 10.1002/mds.1112 11391748

[125] Angus S Sugars J Boltezar R Koskewich S Schneider NM. A controlled trial of amantadine hydrochloride and neuroleptics in the treatment of tardive dyskinesia. J Clin Psychopharmacol (1997) 17(2):88–91. 10.1097/00004714-199704000-00004 10950469

[126] Li CT Chou KH Su TP Huang CC Chen MH Bai YM Gray matter abnormalities in schizophrenia patients with tardive dyskinesia: a magnetic resonance imaging voxel-based morphometry study. PLoS One (2013) 8(8):e71034. 10.1371/journal.pone.0071034 23967150 PMC3744521

[127] Cerasa A Messina D Pugliese P Morelli M Lanza P Salsone M Increased prefrontal volume in PD with levodopa-induced dyskinesias: a voxel-based morphometry study. Mov Disord (2011) 26(5):807–12. 10.1002/mds.23660 21384430

[128] Rascol O Fabre N. Dyskinesia: L-DOPA-induced and tardive dyskinesia. Clin Neuropharmacol (2001) 24(6):313–23. 10.1097/00002826-200111000-00002 11801806

[129] Cerasa A Fasano A Morgante F Koch G Quattrone A. Maladaptive plasticity in levodopa-induced dyskinesias and tardive dyskinesias: old and new insights on the effects of dopamine receptor pharmacology. Front Neurol (2014) 5:49. 10.3389/fneur.2014.00049 24782822 PMC3988357

[130] Greenbaum L Goldwurm S Zozulinsky P Lifschytz T Cohen OS Yahalom G Do tardive dyskinesia and L-DOPA induced dyskinesia share common genetic risk factors? An exploratory study. J Mol Neurosci (2013) 51(2):380–8. 10.1007/s12031-013-0020-x 23666822

[131] Paus S Gadow F Knapp M Klein C Klockgether T Wullner U. Motor complications in patients form the German competence network on Parkinson’s disease and the DRD3 Ser9Gly polymorphism. Mov Disord (2009) 24(7):1080–4. 10.1002/mds.22508 19353703

[132] Foltynie T Cheeran B Williams-Gray CH Edwards MJ Schneider SA Weinberger D BDNF val66met influences time to onset of levodopa induced dyskinesia in Parkinson’s disease. J Neurol Neurosurg Psychiatry (2009) 80(2):141–4. 10.1136/jnnp.2008.154294 18977816

[133] Molchadski I Korczyn AD Cohen OS Katzav A Nitzan Z Chapman J The role of apolipoprotein E polymorphisms in levodopa-induced dyskinesia. Acta Neurol Scand (2011) 123(2):117–21. 10.1111/j.1600-0404.2010.01352.x 21108621

[134] Malhotra AK Murphy GM Jr Kennedy JL. Pharmacogenetics of psychotropic drug response. Am J Psychiatry (2004) 161(5):780–96. 10.1176/appi.ajp.161.5.780 15121641

[135] Arranz MJ Kapur S. Pharmacogenetics in psychiatry: are we ready for widespread clinical use? Schizophr Bull (2008) 34(6):1130–44. 10.1093/schbul/sbn114 18753306 PMC2632495

[136] Ala-Korpela M Kangas AJ Inouye M. Genome-wide association studies and systems biology: together at last. Trends Genet (2011) 27(12):493–8. 10.1016/j.tig.2011.09.002 22018481

[137] Eleftherohorinou H Wright V Hoggart C Hartikainen AL Jarvelin MR Balding D Pathway analysis of GWAS provides new insights into genetic susceptibility to 3 inflammatory diseases. PLoS One (2009) 4(11):e8068. 10.1371/journal.pone.0008068 19956648 PMC2778995

[138] Chahine LM Stern MB Chen-Plotkin A Blood-based biomarkers for Parkinson’s disease. Parkinsonism Relat Disord (2014) 20(Suppl 1):S99–103 10.1016/S1353-8020(13)70025-7 24262199 PMC4070332

[139] Ammal Kaidery N Tarannum S Thomas B. Epigenetic landscape of Parkinson’s disease: emerging role in disease mechanisms and therapeutic modalities. Neurotherapeutics (2013) 10(4):698–708. 10.1007/s13311-013-0211-8 24030213 PMC3805874

